# Supplementary material for: Public and patient perspectives on the use of clinical and administrative health data to identify and contact people at risk of future illness—The case of chronic kidney disease
Source: PLoS One. 2024 Mar 1;19(3):e0298382. doi: 10.1371/journal.pone.0298382 (PMC10906876; doi:10.1371/journal.pone.0298382)
Supplement: S1 Appendix — (PDF) [file pone.0298382.s001.pdf]

Focus Group on Chronic Kidney Disease Case-Finding & Outreach

Don Willison, University of Toronto

Danielle Nash, London Health Sciences Centre &
Lawson Health Research Institute

Agenda

- Opening
  - Goals of meeting
  - Introductions
- The scenario
  - Video: receipt of invitation
  - Responses to video
- Adding context
- Discussion
  - Use of health information for research generally
  - Use of health information for case-finding and contacting
  - Re-visit initial scenario: What would influence your decision to participate?
- Wrap-up

Reminder

- Meeting will be recorded
  - To prepare an anonymized transcript of the conversation
  - If we need to go back to clarify any comments
- We can pause recording at any time.
- Recording will be deleted following preparation of report.

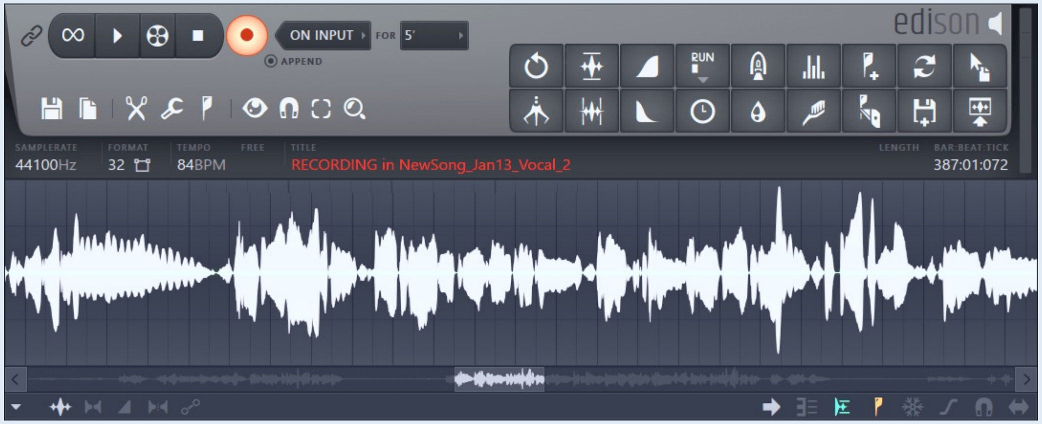

Meeting Ground Rules

- Cameras on
- Microphone off, if not speaking
- Raising of hand
  - If you wish to speak… We will be monitoring for raised hands
  - … if you feel you need to speak to a particular issue that would cause you to jump the queue of people wishing to speak.
- Chat
  - We prefer that you not use this. If you do use it, we may ask you to speak to the topic you have raised.
- CONFIDENTIALITY

Goals of Meeting

To answer the following questions:

1. How acceptable is it to use information that was collected from health services delivery
   - to identify people at risk of serious health conditions who are not receiving appropriate care and
   - to invite them to meet with a doctor for further testing and care?

Goals of Meeting

To answer the following questions:

1. How acceptable is it to use information that was collected from health services delivery
    - to identify people at risk of serious health conditions who are not receiving appropriate care and
    - to invite them to meet with a doctor for further testing and care?
2. How can we maximize participation in such a program?

Introductions

- Moderators
  - Who we are
  - Our sponsors:
    - Canadian Institutes of Health Research through the Canadian Nephrology Trials Network
- Round-table
  - First name
  - What interested you in participating in this focus group?
  - Experience with participating in virtual group meeting

The Scenario

Large Envelope Video - YouTube

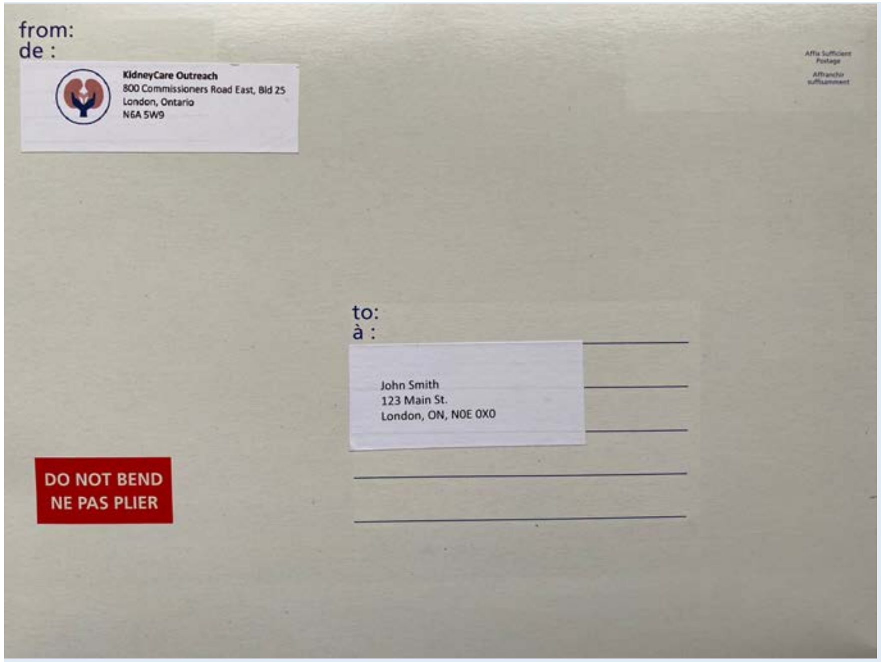
from:
de :
KidneyCare Outreach
800 Commissioners Road East, Bld 25
London, Ontario
N6A 5W9
Affix Sufficient Postage
Affranchir suffisamment
to:
à :
John Smith
123 Main St.
London, ON, N0E 0X0
DO NOT BEND
NE PAS PLIER

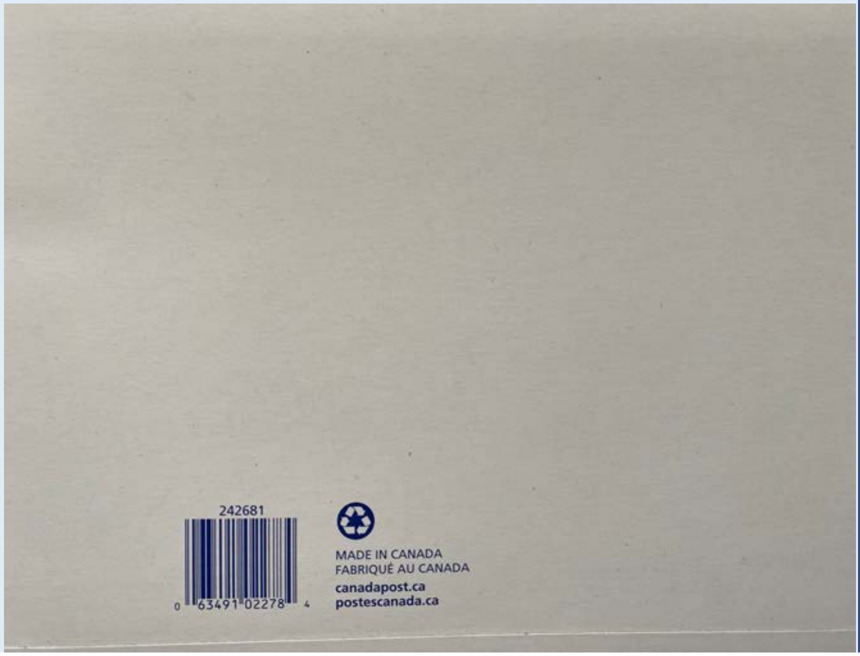
242681
0 63491 02278 4
MADE IN CANADA
FABRIQUÉ AU CANADA
canadapost.ca
postescanada.ca

Discussion

- If you saw this envelope in your mail, what are the chances you would open it?
  - Why? Why not?
- Notes:

- What would make this envelope more inviting to open?
- Notes:

KidneyCare Outreach
www.KidneyOutreach.ca

IC/ES Data Discovery Better Health

PERSONAL AND CONFIDENTIAL

[DATE] Unique ID: [insert ID]

Dear [insert Name of identified individual, e.g., Mr. John Smith],

Kidney disease affects 1 in 10 Canadians. At first, it has no symptoms. But left untreated, it can progress to needing life-sustaining treatment that can limit a person's quality-of-life. Kidney disease also increases the risk for other serious problems, like high blood pressure, heart attack and stroke. The good news is that when kidney problems are found early, medications can be given to slow down or stop the loss of kidney function.

Based on recent lab tests that you had done on [insert date],
your kidney test results suggest the need for further attention.

KidneyCare Outreach is a new initiative in Ontario. When Ontarians use OHIP-funded health services (e.g., lab testing, doctors' visits), data from these visits are routinely collected by the Ontario Ministry of Health. These data are held at ICES (https://ices.on.ca), a not-for-profit research institute that analyzes data to improve health care policy. ICES data were used to identify individuals, like yourself, who may be candidates for a kidney health outreach initiative. ***Your information has always been kept secure and protected*** under the Personal Health Information Protection Act, the law which governs the collection, use and disclosure of personal health information in Ontario.

Because KidneyCare Outreach is new, it is being launched as a research study to learn how to best connect with people who may be living with kidney disease. Just like how Ontario offers cancer screening tests to try to detect cancer earlier, the goal of KidneyCare Outreach is to ensure that persons at risk of progressive kidney disease have access to the best possible care.

The purpose of this letter is to get your consent to be contacted by the KidneyCare Outreach research team to tell you more about the initiative. ***If you choose to participate, you will get updated lab testing and have a conversation with a kidney doctor***. There are no costs to participate. Following this, the research team may connect you with kidney care that you might not have received otherwise. More details can be found in the 'Frequently Asked Questions' on the next page and in the information pamphlet from the Kidney Foundation.

To learn more about how to take the next step to protect your kidney health, visit www.KidneyOutreach.ca or call 1.888.584.8882 today. If we do not hear from you, an ICES representative may phone you to follow-up a few weeks after you receive this letter.

Sincerely,

Dr. Amit Garg, MD
ICES Senior Core Scientist
Kidney Specialist, London Health Sciences Centre

Dr. Ann Young, MD
ICES Fellow
Kidney Specialist, Unity Health - Toronto

FREQUENTLY ASKED QUESTIONS

This is the first time I am hearing about possible problems with my kidneys. Why is this information not coming from my family doctor?
You were identified as a person who may be living with kidney disease based on a recent lab test that you had done. This test may not have been ordered by your regular family doctor. Some people may not have a regular family doctor. Kidney disease is a complex health condition and sometimes important indicators of kidney health may be missed. Also, in a complex health system, sometimes even important issues can 'fall through the cracks'. Kidney Care Outreach was designed to **support health care providers** to make sure fewer patients are overlooked, kind of like a "safety net". We would be happy to connect with your family doctor about your kidney health, but this can only be done after we get your consent.

Why should I participate in this study instead of going directly to my family doctor?
As a person who may be living with kidney disease, it is important to have your kidney health assessed – this can be done either through the study or through your own family doctor. Our primary interest is in making sure that you receive the best kidney care possible. Participating in the study will allow us to ensure that you are appropriately connected with the care you need. It will also allow us to collect new data that can help improve the program so that we can help other people who may also be living with kidney disease but not know it. With your consent, we can start communicating with your family doctor and all work together.

What is ICES?
ICES is a not-for-profit research institute in Ontario, funded by the Ministry of Health, leads cutting-edge studies evaluating health care delivery and outcomes. Our researchers access a vast and secure array of Ontario's health-related data. ICES is recognized as a leader in maintaining the privacy and security of health information. For further information, please go to the ICES website (https://www.ices.on.ca/).

I am concerned about my privacy. How does ICES have access to my personal health information?
ICES has special status under Ontario's Personal Health Information Protection Act (PHIPA) as a "prescribed entity", which allows ICES access to routinely-collected personal health information gathered during the provision of health care services across Ontario without patients' explicit consent. ICES uses this information to evaluate and monitor the health system to improve quality and efficiency of care, and to conduct research projects approved by a research ethics board. Also, ICES' policies, practices, and procedures are reviewed on a regular basis by the Office of the Information Privacy Commissioner of Ontario, which oversees the use of personal information (https://www.ipc.on.ca/). For further information, please visit the Privacy at ICES webpage https://www.ices.on.ca/Data-and-Privacy/Privacy-at-ICES).

What if I do not want to be contacted by the Kidney Care Outreach Program?
To stop receiving letters, fill out the Opt Out form at www.KidneyOutreach.ca/opt-out or call 1.888.584.8882. If you decide not to participate, the only information that our research team will collect about you is that you chose not to participate in the study. The Ontario Ministry of Health Data will continue to routinely collect data on publicly funded health services to evaluate and monitor the health system. Data for such analyses are de-identified and presented in aggregated form (i.e., you cannot be individually identified).
You may change your mind at any time by reconnecting with us.

For answers to additional questions, please visit www.KidneyOutreach.ca.

PERSONAL AND CONFIDENTIAL

[DATE] Unique ID: [insert ID]

Dear [insert Name of identified individual, e.g., Mr. John Smith],

Kidney disease affects 1 in 10 Canadians. At first, it has no symptoms. But left untreated, it can progress to needing life-sustaining treatment that can limit a person's quality-of-life. Kidney disease also increases the risk for other serious problems, like high blood pressure, heart attack and stroke. The good news is that when kidney problems are found early, medications can be given to slow down or stop the loss of kidney function.

> Based on recent lab tests that you had done on [insert date],
> **your kidney test results suggest the need for further attention.**

KidneyCare Outreach is a new initiative in Ontario. When Ontarians use OHIP-funded health services (e.g., lab testing, doctors' visits), data from these visits are routinely collected by the Ontario Ministry of Health. These data are held at ICES (https://ices.on.ca), a not-for-profit research institute that analyzes data to improve health care policy. ICES data were used to identify individuals, like yourself, who may be candidates for a kidney health outreach initiative. ***Your information has always been kept secure and protected*** under the Personal Health Information Protection Act, the law which governs the collection, use and disclosure of personal health information in Ontario.

Because KidneyCare Outreach is new, it is being launched as a research study to learn how to best connect with people who may be living with kidney disease. Just like how Ontario offers cancer screening tests to try to detect cancer earlier, the goal of KidneyCare Outreach is to ensure that persons at risk of progressive kidney disease have access to the best possible care.

The purpose of this letter is to get your consent to be contacted by the KidneyCare Outreach research team to tell you more about the initiative. ***If you choose to participate, you will get updated lab testing and have a conversation with a kidney doctor***. There are no costs to participate. Following this, the research team may connect you with kidney care that you might not have received otherwise. More details can be found in the 'Frequently Asked Questions' on the next page and in the information pamphlet from the Kidney Foundation.

To learn more about how to take the next step to protect your kidney health, visit www.KidneyOutreach.ca or call 1.888.584.8882 today. If we do not hear from you, an ICES representative may phone you to follow-up a few weeks after you receive this letter.

Discussion

- What do you think this letter is asking you to do?
- Notes:

Discussion

- What do you think this letter is asking you to do?
- What is going through your mind as you read this?
- Notes:

Discussion

- What do you think this letter is asking you to do?
- What is going through your mind as you read this?
- How would you respond to this invitation?
- Notes:

Feedback on specific revisions to the letter

- If the letter had a logo from the Kidney Foundation, would this influence your response to the letter?

KidneyCare Outreach
www.KidneyOutreach.ca

IC/ES Data Discovery Better Health

kidney FOUNDATION

PERSONAL AND CONFIDENTIAL

[DATE] Unique ID: [insert ID]

Dear [insert Name of identified individual, e.g., Mr. John Smith],

- Notes:

Feedback on specific revisions to the letter

- What are your thoughts on the wording in the last paragraph of the letter starting with 'If we do not hear from you...'?

To learn more about how to take the next step to protect your kidney health, visit www.KidneyOutreach.ca or call 1.888.584.8882 today. If we do not hear from you, an ICES representative may phone you to follow-up a few weeks after you receive this letter.

Sincerely,

- Does this sentence make it more or less likely that you would respond to the letter?
- Notes:

FREQUENTLY ASKED QUESTIONS

This is the first time I am hearing about possible problems with my kidneys. Why is this information not coming from my family doctor?

You were identified as a person who may be living with kidney disease based on a recent lab test that you had done. This test may not have been ordered by your regular family doctor. Some people may not have a regular family doctor. Kidney disease is a complex health condition and sometimes important indicators of kidney health may be missed. Also, in a complex health system, sometimes even important issues can 'fall through the cracks'. Kidney Care Outreach was designed to **support health care providers** to make sure fewer patients are overlooked, kind of like a "safety net". We would be happy to connect with your family doctor about your kidney health, but this can only be done after we get your consent.

Why should I participate in this study instead of going directly to my family doctor?

As a person who may be living with kidney disease, it is important to have your kidney health assessed – this can be done either through the study or through your own family doctor. Our primary interest is in making sure that you receive the best kidney care possible. Participating in the study will allow us to ensure that you are appropriately connected with the care you need. It will also allow us to collect new data that can help improve the program so that we can help other people who may also be living with kidney disease but not know it. With your consent, we can start communicating with your family doctor and all work together.

What is ICES?
ICES is a not-for-profit research institute in Ontario, funded by the Ministry of Health, leads cutting-edge studies evaluating health care delivery and outcomes. Our researchers access a vast and secure array of Ontario's health-related data. ICES is recognized as a leader in maintaining the privacy and security of health information. For further information, please go to the ICES website (https://www.ices.on.ca/).

I am concerned about my privacy. How does ICES have access to my personal health information?
ICES has special status under Ontario's Personal Health Information Protection Act (PHIPA) as a "prescribed entity", which allows ICES access to routinely-collected personal health information gathered during the provision of health care services across Ontario without patients' explicit consent. ICES uses this information to evaluate and monitor the health system to improve quality and efficiency of care, and to conduct research projects approved by a research ethics board. Also, ICES' policies, practices, and procedures are reviewed on a regular basis by the Office of the Information Privacy Commissioner of Ontario, which oversees the use of personal information (https://www.ipc.on.ca/). For further information, please visit the Privacy at ICES webpage https://www.ices.on.ca/Data-and-Privacy/Privacy-at-ICES).

What if I do not want to be contacted by the Kidney Care Outreach Program?
To stop receiving letters, fill out the Opt Out form at www.KidneyOutreach.ca/opt-out or call 1.888.584.8882. If you decide not to participate, the only information that our research team will collect about you is that you chose not to participate in the study. The Ontario Ministry of Health Data will continue to routinely collect data on publicly funded health services to evaluate and monitor the health system. Data for such analyses are de-identified and presented in aggregated form (i.e., you cannot be individually identified).
You may change your mind at any time by reconnecting with us.

For answers to additional questions, please visit www.KidneyOutreach.ca.

Discussion

Adding Context

Use of health information for research and quality improvement

How does information collected during health care end up being used to improve the health system?

- Is anyone familiar with ICES (formerly the Institute for Clinical Evaluative Sciences)?
- https://www.youtube.com/watch?v=GqP_ydqk6yo
- Questions / Responses to the video?

Discussion

- Prior to this focus group meeting, were you aware that health information collected for health services delivery is being used in these ways?
- Notes:

Who/What is ICES?

- Who?
  - Independent not-for-profit research institute
    - Researchers, data and policy experts, health care experts
  - Established in 1992
  - Special legal status to work with health data collected in the process of providing patient care (called a “prescribed entity”)

Who/What is ICES?

- Who?
  - Independent not-for-profit research institute
    - Researchers, data and policy experts, health care experts
  - Established in 1992
  - Special legal status to work with health data collected in the process of providing patient care (called a "prescribed entity")
- What do they do?
  - Research on health care delivery and health outcomes
  - Compile statistics for planning and management of health care system
  - Use data from:
    - Administrative databases (physician billing, hospital records, prescriptions dispensed)
    - Lab tests (Ontario Lab Information System)
    - Other (e.g., anonymized patient records, health surveys)

ICES (cont.)

- Governance
  - Law: Personal Health Information Protection Act (PHIPA)
  - Contracts with government and other data holders
  - Reviewed by Ontario Information and Privacy Commissioner every 3 years

ICES (cont.)

- Governance
  - Law: Personal Health Information Protection Act (PHIPA)
  - Contracts with government and other data holders
  - Reviewed by Ontario Information and Privacy Commissioner every 3 years
- Privacy and security protections
  - Physical (e.g., locked doors, cameras)
  - Technical (e.g., data handling, no way of taking data out of system)
  - Procedural (e.g., restricts who has access (role-based access))
  - Other (e.g., replaces identifying information, such as name and health card number, with a unique, anonymous identifier)

ICES (cont.)

- Works with various partners
  - Researchers at universities
  - Health care providers at hospitals
  - Ontario government agencies (e.g., Ontario Health including Cancer Care Ontario and Ontario Renal Network)
  - Canadian Institute for Health Information
  - Indigenous organizations
  - Etc.

Discussion

- How do you feel about ICES using health data collected from services that you have used [as in the video you just saw] for:
  - managing the healthcare system to improve health outcomes?
  - heath research?

  [Considering the safeguards described in the video]
- Notes:

“New” Use of Data from ICES for Case-finding and Contacting: KidneyCare Outreach

Chronic Kidney Disease

Burden

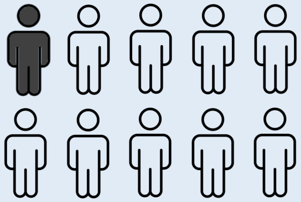

10%

~4 Million Canadians

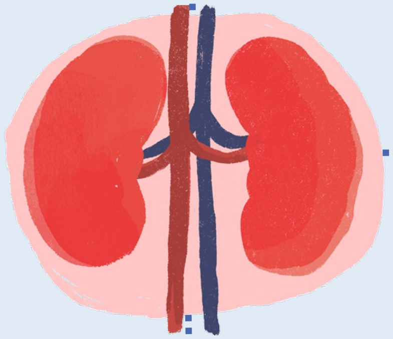

Chronic Kidney Disease

Burden

10%

~4 Million Canadians

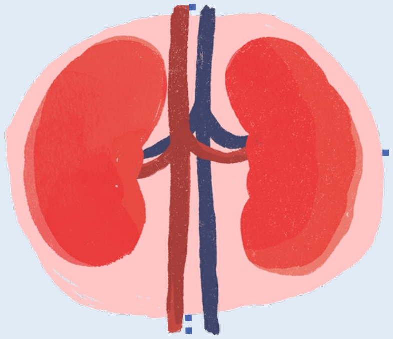

Kidney Failure

> 50,000

Canadians

in 2019

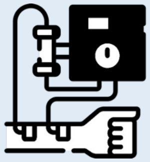

Chronic Kidney Disease

Burden

10%
~4 Million
Canadians

Kidney Failure

> 50,000
Canadians
in 2019

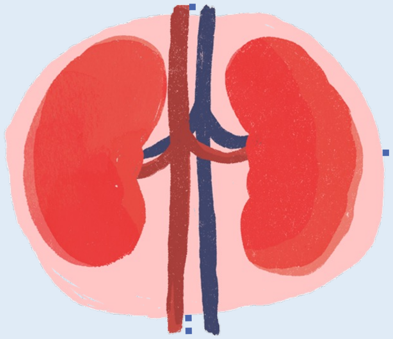

Severity

10th
leading cause of death
in Canada in 2018

Chronic Kidney Disease

Burden

10%

~4 Million Canadians

Kidney Failure

> 50,000

Canadians

in 2019

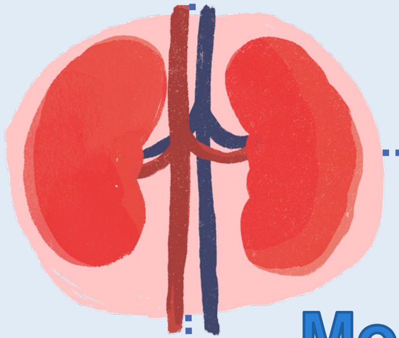

Severity

10th

leading cause of death

in Canada in 2018

Late Referrals

More than 25%

of patients starting dialysis saw a kidney specialist for <3 months prior

Chronic Kidney Disease Screening

- Identifying CKD is not difficult
- It can be diagnosed using two lab tests:

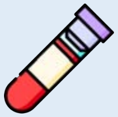

  - a blood test for creatinine, to estimate level of kidney function, and

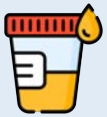

  - a urine test for the albumin-to-creatinine ratio (ACR) – a marker of kidney damage
- These tests can be ordered by a family doctor / nurse practitioner and done at any laboratory in Ontario

For years…

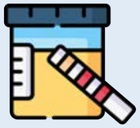

1. Researchers have performed studies using all kidney function tests in Ontario

For years…

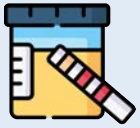

1. Researchers have performed studies using all kidney function tests in Ontario

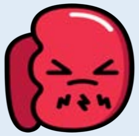

2. Researchers know there are at-risk people who aren’t getting the kidney care they need, and

For years…

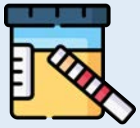

1. Researchers have performed studies using all kidney function tests in Ontario

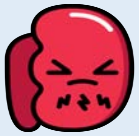

2. Researchers know there are at-risk people who aren't getting the kidney care they need, and

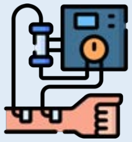

3. Nephrologists only become aware of these people after they have reached kidney failure

For years…

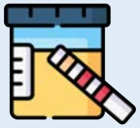

1. Researchers have performed studies using all kidney function tests in Ontario

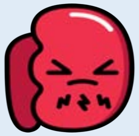

2. Researchers know there are at-risk people who aren't getting the kidney care they need, and

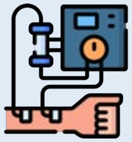

3. Nephrologists only become aware of these people after they have reached kidney failure

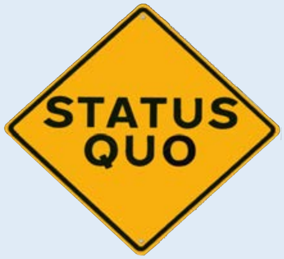

Up to now, groups like ICES could use the data for research so long as they did not contact individuals in the dataset.

A time of transition?

- Regulators are interested in learning whether there are times when it may be OK to contact people:
  - At high risk for (serious) illness
  - Not yet receiving care (preventative or treatment)

- **That is what we want to explore in this focus group**
  - How acceptable is the proposed outreach plan? (See next slide.)
    - Approval received by the Information and Privacy Commissioner of Ontario and Western Research Ethics Board

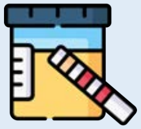

People receive routine laboratory testing

→

Healthcare data held at ICES

→

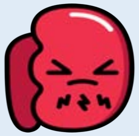

Data shows people at high risk for kidney failure not seeing a kidney specialist

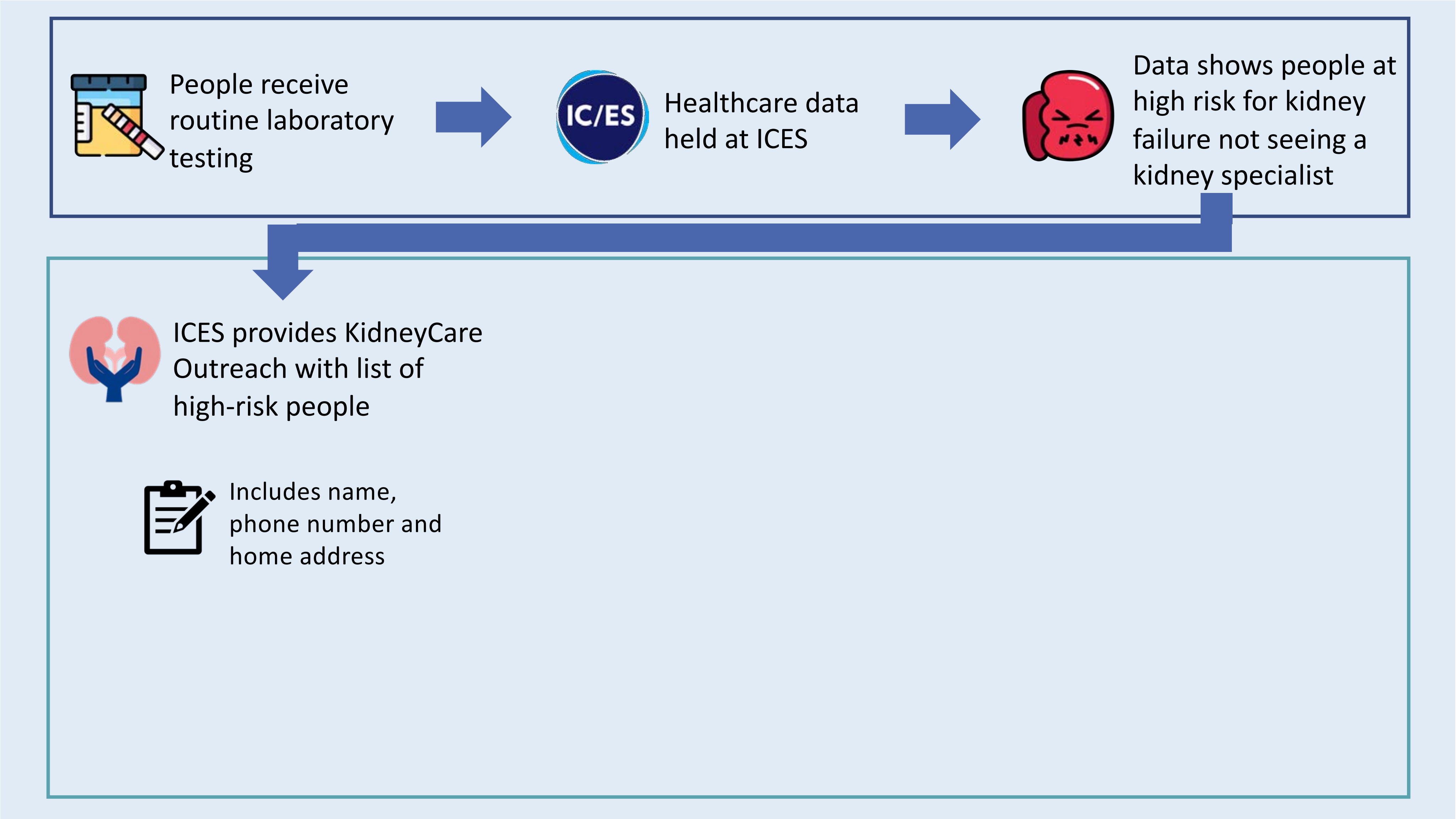
People receive routine laboratory testing
IC/ES
Healthcare data held at ICES
Data shows people at high risk for kidney failure not seeing a kidney specialist
ICES provides KidneyCare Outreach with list of high-risk people
Includes name, phone number and home address

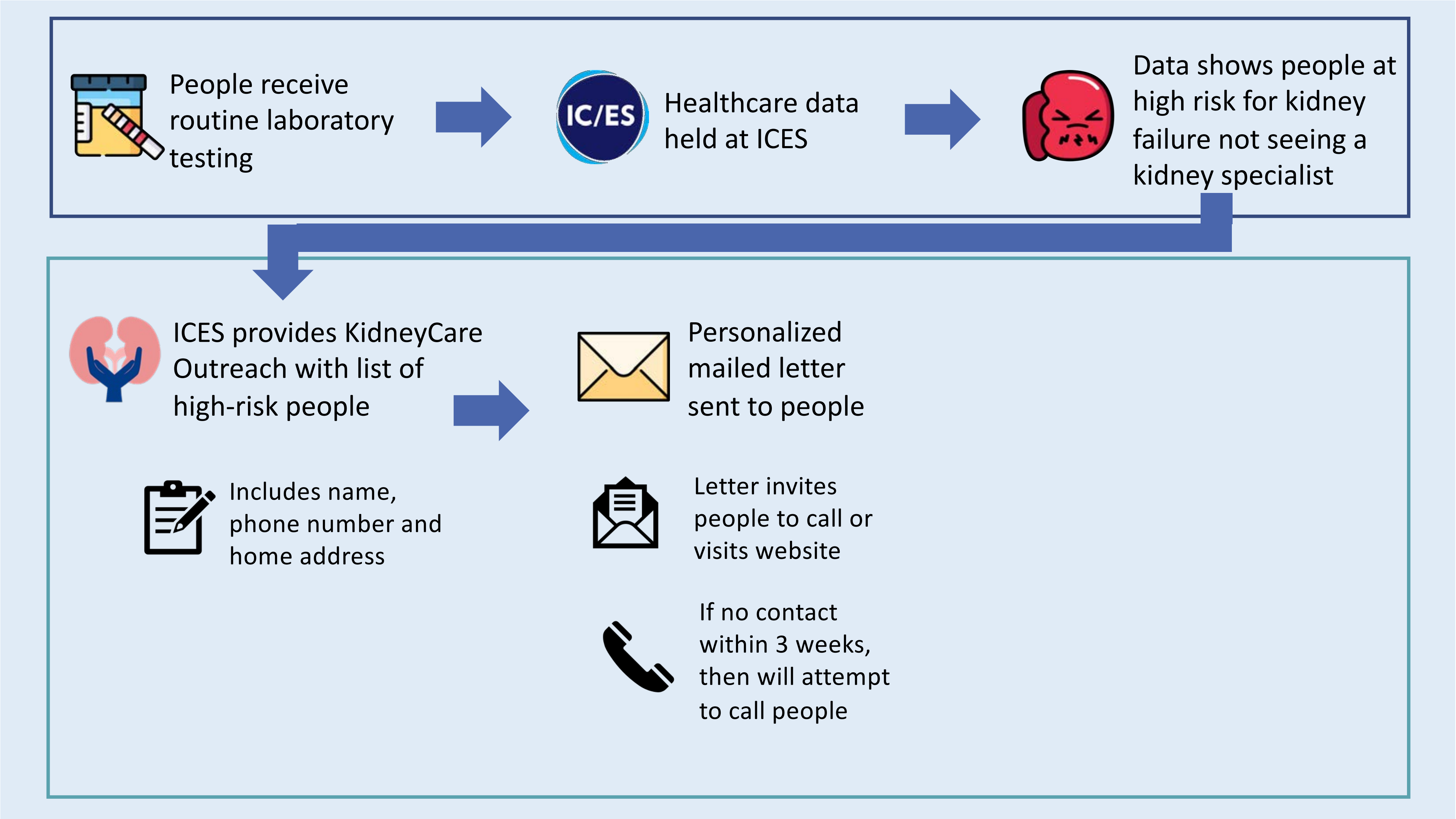
IC/ES
People receive routine laboratory testing
Healthcare data held at ICES
Data shows people at high risk for kidney failure not seeing a kidney specialist
ICES provides KidneyCare Outreach with list of high-risk people
Personalized mailed letter sent to people
Includes name, phone number and home address
Letter invites people to call or visits website
If no contact within 3 weeks, then will attempt to call people

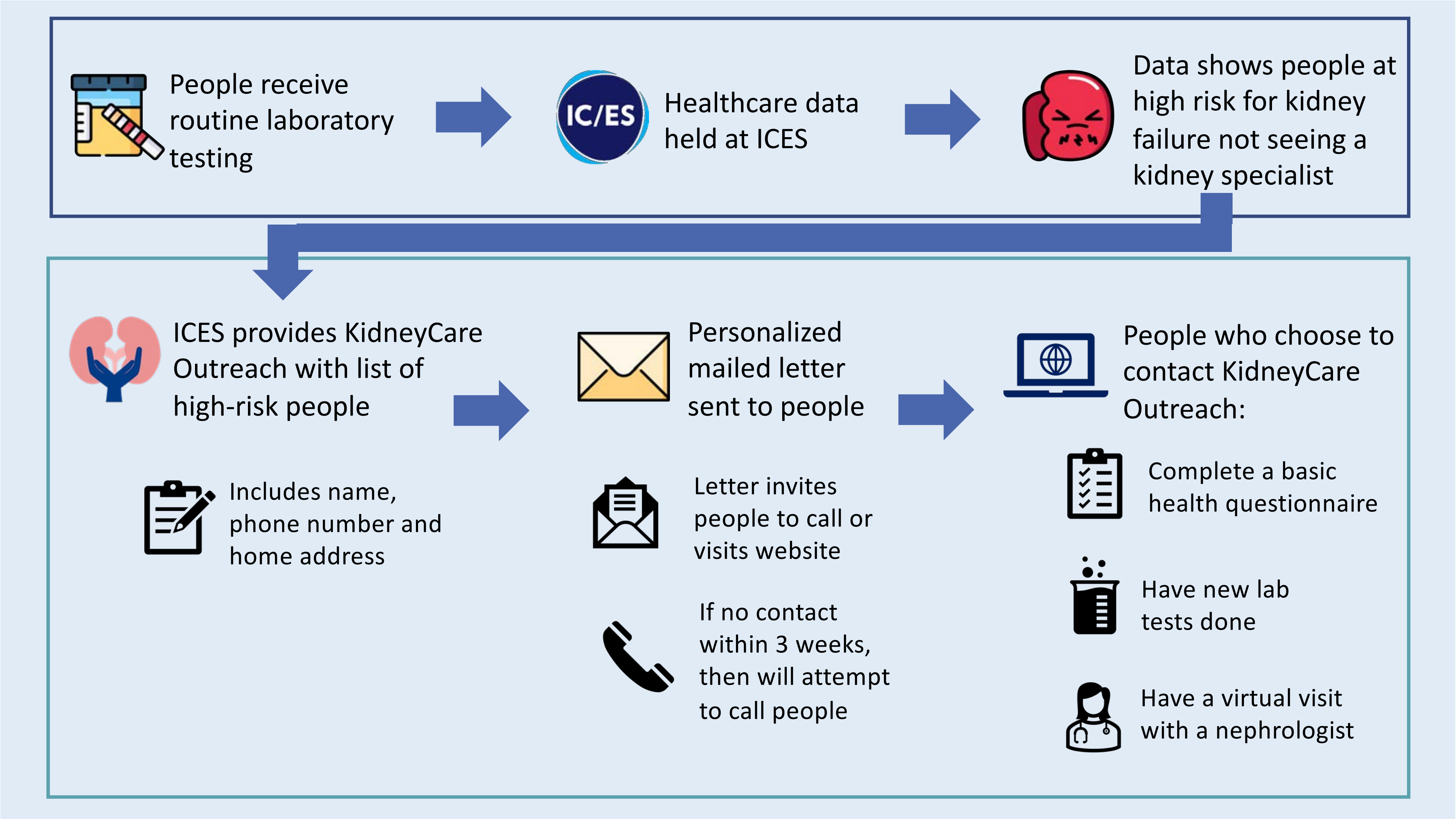
People receive routine laboratory testing
IC/ES
Healthcare data held at ICES
Data shows people at high risk for kidney failure not seeing a kidney specialist
ICES provides KidneyCare Outreach with list of high-risk people
Includes name, phone number and home address
Personalized mailed letter sent to people
Letter invites people to call or visits website
If no contact within 3 weeks, then will attempt to call people
People who choose to contact KidneyCare Outreach:
Complete a basic health questionnaire
Have new lab tests done
Have a virtual visit with a nephrologist

Overall Goal of KidneyCare Outreach

Set up a provincial 'safety net' with kidney care outreach so 'at risk' people:

- are seen in specialized kidney clinics,
- receive the care they need to help delay progression to kidney failure, and
- receive information to make decisions about any future treatments.

Discussion

- Do you have questions about what KidneyCare Outreach wants to do or how they propose to do it?
- Thoughts?
- Notes:

Precedent: Cancer Care Ontario

- Letters sent out to Ontarians who are collectively at increased risk (based on age and sex) for:
  - colon cancer
  - breast cancer
  - cervical cancer

- Who here is aware of this screening program?
- Has anyone here been contacted by CCO?
  - Did you respond?

What do you think of this CCO service?

- Notes:

Back to the Chronic Kidney Disease Scenario

- What do you like about what KidneyCare Outreach wants to do?
- Do you have any concerns?
- Notes:

- What do you think about an organization you don’t know (in this case KidneyCare Outreach) contacting people directly by letter?
  - Notes:

- What (if anything) would make this more acceptable?
  - Notes:

Returning to the opening scenario…

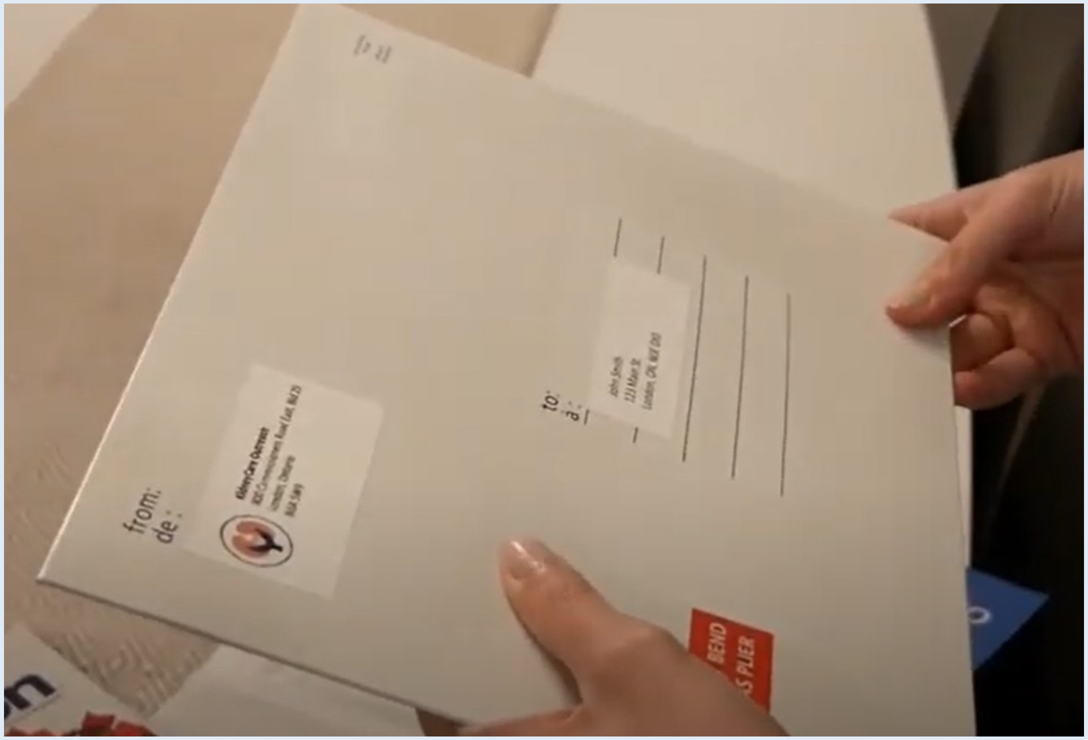

In light of our discussion…

- Now, what do you think of the invitation letter?
- Notes:

KidneyCare Outreach
www.KidneyOutreach.ca

IC/ES Data Discovery Better Health

PERSONAL AND CONFIDENTIAL

[DATE] Unique ID: [insert ID]

Dear [insert Name of identified individual, e.g., Mr. John Smith],

Kidney disease affects 1 in 10 Canadians. At first, it has no symptoms. But left untreated, it can progress to needing life-sustaining treatment that can limit a person's quality-of-life. Kidney disease also increases the risk for other serious problems, like high blood pressure, heart attack and stroke. The good news is that when kidney problems are found early, medications can be given to slow down or stop the loss of kidney function.

Based on recent lab tests that you had done on [insert date],
your kidney test results suggest the need for further attention.

KidneyCare Outreach is a new initiative in Ontario. When Ontarians use OHIP-funded health services (e.g., lab testing, doctors' visits), data from these visits are routinely collected by the Ontario Ministry of Health. These data are held at ICES (https://ices.on.ca), a not-for-profit research institute that analyzes data to improve health care policy. ICES data were used to identify individuals, like yourself, who may be candidates for a kidney health outreach initiative. ***Your information has always been kept secure and protected*** under the Personal Health Information Protection Act, the law which governs the collection, use and disclosure of personal health information in Ontario.

Because KidneyCare Outreach is new, it is being launched as a research study to learn how to best connect with people who may be living with kidney disease. Just like how Ontario offers cancer screening tests to try to detect cancer earlier, the goal of KidneyCare Outreach is to ensure that persons at risk of progressive kidney disease have access to the best possible care.

The purpose of this letter is to get your consent to be contacted by the KidneyCare Outreach research team to tell you more about the initiative. ***If you choose to participate, you will get updated lab testing and have a conversation with a kidney doctor***. There are no costs to participate. Following this, the research team may connect you with kidney care that you might not have received otherwise. More details can be found in the 'Frequently Asked Questions' on the next page and in the information pamphlet from the Kidney Foundation.

To learn more about how to take the next step to protect your kidney health, visit www.KidneyOutreach.ca or call 1.888.584.8882 today. If we do not hear from you, an ICES representative may phone you to follow-up a few weeks after you receive this letter.

Sincerely,

Dr. Amit Garg, MD
ICES Senior Core Scientist
Kidney Specialist, London Health Sciences Centre

Dr. Ann Young, MD
ICES Fellow
Kidney Specialist, Unity Health - Toronto

In light of our discussion…

- How could we make it more likely:
  - That you would open the letter?
  - Notes:

  - That you would respond to the letter?
  - Notes:

- What might influence you to not respond?
  - Notes:

What if it was a less serious but still treatable condition being addressed?

- How would it affect your decision to participate? Why?

- Notes:

What if there was no way to prevent the outcome through early detection?

- How would it affect your decision to participate? Why?

- Notes:

If this kind of case finding and outreach were to expand to a wide variety of health conditions, how would that affect your response to receiving these letters?

- Notes:

As an initial step before this can become routine practice, a research phase is needed (i.e., a smaller number of people would be contacted and invited to participate as a research study).

Would this change your view or opinion?

- Notes:

Up to now, we have been asking about the acceptability of being contacted directly by KidneyCare Outreach.

Let’s now consider, if:

(a) The Province has the ability to identify specific people in its databases who are at high risk of a life-threatening illness, and

(b) That illness can be treated or prevented if caught early enough

Do you think there is an ethical imperative / social responsibility to notify people?

- Notes:

That's it for our questions. Do you have any further questions of us?

- Notes:

Thank you!
