# Supplementary material for: Public and patient perspectives on the use of clinical and administrative health data to identify and contact people at risk of future illness—The case of chronic kidney disease
Source: PLoS One. 2024 Mar 1;19(3):e0298382. doi: 10.1371/journal.pone.0298382 (PMC10906876; doi:10.1371/journal.pone.0298382)
Supplement: S2 Appendix — Note this is the second version of the letter presented to the last three focus groups. (DOCX) [file pone.0298382.s002.docx]

**Personal and Confidential**

[DATE] Unique ID: [insert ID]

Dear [insert Name of identified individual, e.g., Mr. John Smith],

Kidney disease affects 1 in 10 Canadians. At first, it has no symptoms. But left untreated, it can progress to needing life-sustaining treatment that can limit a person’s quality-of-life. Kidney disease also increases the risk for other serious problems, like high blood pressure, heart attack and stroke. The good news is that when kidney problems are found early, medications can be given to slow down or stop the loss of kidney function.

Based on recent lab tests that you had done on [insert date], **your kidney test results suggest the need for further attention.**

KidneyCare Outreach is a new initiative in Ontario. When Ontarians use OHIP-funded health services (e.g., lab testing, doctors’ visits), data from these visits are routinely collected by the Ontario Ministry of Health. These data are held at ICES (<https://ices.on.ca>), a not-for-profit research institute that analyzes data to improve health care policy. ICES data were used to identify individuals, like yourself, who may be candidates for a kidney health outreach initiative. ***Your information has always been kept secure and protected*** under the Personal Health Information Protection Act, the law which governs the collection, use and disclosure of personal health information in Ontario.

Because KidneyCare Outreach is new, it is being launched as a research study to learn how to best connect with people who may be living with kidney disease. Just like how Ontario offers cancer screening tests to try to detect cancer earlier, the goal of KidneyCare Outreach is to ensure that persons at risk of progressive kidney disease have access to the best possible care.

The purpose of this letter is to get your consent to be contacted by the KidneyCare Outreach research team to tell you more about the initiative. ***If you choose to participate, you will get updated lab testing and have a conversation with a kidney doctor***. There are no costs to participate. Following this, the research team may connect you with kidney care that you might not have received otherwise. More details can be found in the ‘Frequently Asked Questions’ on the next page and in the information pamphlet from the Kidney Foundation.

To learn more about how to take the next step to protect your kidney health, visit [www.KidneyOutreach.ca](http://www.KidneyOutreach.ca) or call 1.888.584.8882 today. If we do not hear from you, an ICES representative may phone you to follow-up a few weeks after you receive this letter.

Sincerely,

Dr. Amit Garg, MD Dr. Ann Young, MD

ICES Senior Core Scientist ICES Fellow

Kidney Specialist, London Health Sciences Centre Kidney Specialist, Unity Health - Toronto

**FREQUENTLY ASKED QUESTIONS**

**This is the first time I am hearing about possible problems with my kidneys. Why is this information not coming from my family doctor?**

You were identified as a person who may be living with kidney disease based on a recent lab test that you had done. This test may not have been ordered by your regular family doctor. Some people may not have a regular family doctor. Kidney disease is a complex health condition and sometimes important indicators of kidney health may be missed. Also, in a complex health system, sometimes even important issues can ‘fall through the cracks’. Kidney Care Outreach was designed to **support health care providers** to make sure fewer patients are overlooked, kind of like a “safety net”. We would be happy to connect with your family doctor about your kidney health, but this can only be done after we get your consent.

**Why should I participate in this study instead of going directly to my family doctor?**

As a person who may be living with kidney disease, it is important to have your kidney health assessed – this can be done either through the study or through your own family doctor. Our primary interest is in making sure that you receive the best kidney care possible. Participating in the study will allow us to ensure that you are appropriately connected with the care you need. It will also allow us to collect new data that can help improve the program so that we can help other people who may also be living with kidney disease but not know it. With your consent, we can start communicating with your family doctor and all work together.

**What is ICES?**

ICES is a not-for-profit research institute in Ontario, funded by the Ministry of Health, leads cutting-edge studies evaluating health care delivery and outcomes. Our researchers access a vast and secure array of Ontario’s health-related data. ICES is recognized as a leader in maintaining the privacy and security of health information. For further information, please go to the ICES website (<https://www.ices.on.ca/>).

**I am concerned about my privacy. How does ICES have access to my personal health information?**

ICES has special status under Ontario's Personal Health Information Protection Act (PHIPA) as a “prescribed entity”, which allows ICES access to routinely-collected personal health information gathered during the provision of health care services across Ontario without patients’ explicit consent. ICES uses this information to evaluate and monitor the health system to improve quality and efficiency of care, and to conduct research projects approved by a research ethics board. Also, ICES’ policies, practices, and procedures are reviewed on a regular basis by the Office of the Information Privacy Commissioner of Ontario, which oversees the use of personal information (<https://www.ipc.on.ca/>). For further information, please visit the Privacy at ICES webpage <https://www.ices.on.ca/Data-and-Privacy/Privacy-at-ICES>).

**What if I do not want to be contacted by the Kidney Care Outreach Program?**

To stop receiving letters, fill out the Opt Out form at www.KidneyOutreach.ca/opt-out or call 1.888.584.8882. If you decide not to participate, the only information that our research team will collect about you is that you chose not to participate in the study. The Ontario Ministry of Health Data will continue to routinely collect data on publicly funded health services to evaluate and monitor the health system. Data for such analyses are de-identified and presented in aggregated form (i.e., you cannot be individually identified).

You may change your mind at any time by reconnecting with us.

**For answers to additional questions, please visit www.KidneyOutreach.ca.**
