# Supplementary material for: Public and patient perspectives on the use of clinical and administrative health data to identify and contact people at risk of future illness—The case of chronic kidney disease
Source: PLoS One. 2024 Mar 1;19(3):e0298382. doi: 10.1371/journal.pone.0298382 (PMC10906876; doi:10.1371/journal.pone.0298382)
Supplement: S3 Appendix — (DOCX) [file pone.0298382.s003.docx]

Thematic summary of focus group findings

**Notes:**

Text in blue reflects direct quotes

Text in red reflects comments from DJW and DMN

| **Summary of Narrative** | **Raised in Group** | |
| --- | --- | --- |
|  | **CKD** | **GP** |
| **1 What would improve participation in study?** |  |  |
| **1.1 The envelope** |  |  |
| Generally, most would open it | ALL | ALL |
| 1. Curiosity | CKD1 | ALL |
| - *‘Do not bend’ piques interest* | CKD1, CKD3 | GP2 |
| 1. Looks official and legitimate (not worried it’s a scam or spam) for the following reasons: | ALL | ALL |
| - Addressed to them | CKD1, CKD3 | ALL |
| - Look of envelope (size, cardboard, not colourful, no advertising) | ALL | ALL |
| - “If something’s cardboard or official like that, it makes it in my mind feel like it’s more important and it matters more” - [CKD2 (at 24:12)] | CKD2 | - |
| 1. Because it is from a kidney organization (however don’t recognize organization) | ALL | - |
| 1. No danger in opening it (as compared to email): “OK, if I received this in the mail, I would open it because there's no danger of opening it. If I receive that as an e-mail, it would go to trash immediately.” – [GP1 (R1, at 17:15)] | - | GP1 |
| 1. One person would not open it because they get a lot of junk mail from fundraisers | - | GP2 |
|  |  |  |
| **1.2 The letter / message itself** |  |  |
| Overall, positive feedback | ALL | ALL |
| 1. Professional looking |  |  |
| - Liked the signatures of the doctors at the end with their organizations (however, people may not be familiar with these hospital names) | - | GP2 |
| - Kidney Foundation logo adds credibility to the letter   [This logo was added to the letter in response to feedback in the first three focus groups, and we specifically solicited feedback about this in CKD3, GP2, and GP3 focus groups] | CKD3 | GP2, GP3 |
| 1. The box with the lab information immediately caught their attention | - | GP2, GP3 |
| Most people understood the message in the letter: “One it's telling you that they identified something of concern and second the organization is asking your consent to get involved.” - [GP3 (at 32:50)] | ALL | ALL |
| 1. Some concern that the letter is difficult to understand if English not first language [CKD1]: “I think here you've got too many big words for some people because not everyone has English as their first language and it's a complex subject and some people need someone to interpret this for them because they don't have any English. So in terms of equity, it's not really hitting me really well there either.” [CKD1 (at 40:50)] | CKD1 | - |
|  |  |  |
| Need to find balance between informing people there is a concern and not alarming them   1. Gentle alert – wording in letter that they “may have CKD” was found to be alarming but after this was changed in the letter participants thought it was a gentler way to inform them of a potential problem.  - “The letter is very alarming” [CKD2 (Linda 33:53)] - The box with the lab information immediately caught attention. Was difficult to move onto the rest of the letter after that.   Note we changed this wording in the letter for focus groups GP2, GP3 and CKD3   - “It wasn't alarming. It was very gentle, informative, it sort of answered and anticipated all the questions I would have had if I were in that scenario.” [GP3 (R3, at 34:52)]   This comment was in response to the revised letter | CKD2 | GP2, GP3 |
| Would want reassurance about privacy and security; some concerns about privacy breaches lately | - | GP2, GP3 |
| 1. Make it clearer how the organization got their personal information | CKD1, CKD2 | GP1, GP3 |
|  |  |  |
| **1.3 The messenger** |  |  |
| Messenger should be a recognized, credible source | CKD1, CKD2 | ALL |
| 1. Curious how the organization got their information | CKD1, CKD2 | GP1, GP3 |
| 1. Need legitimacy / connected with healthcare system or government | CKD1, CKD2 | GP2, GP3 |
|  |  |  |
| Prefer message to come from the provider who ordered the test | ALL | ALL |
| 1. Trust doctor vs. unknown organization: “It comes down to a matter of trust. I've been with my family doctor for 16 years, and I trust that source of information versus some organization I have not heard from. So they got to work together. You can't have 2 conflicting viewpoints.” - [CKD3 (R2 at 2:04:18)] | CKD3 | - |
| 1. Should be given by trusted healthcare professional who you can ask questions to at the time (especially for more serious illnesses) | CKD2, CKD3 | - |
| 1. Some participants recognized that this may not be feasible given that healthcare providers are already overworked and that some things get missed and that some people don’t have a family doctor | - | GP2 |
|  |  |  |
| **1.4 The process** |  |  |
| There should be a general notice to the public indicating their information may be used to identify and contact at-risk individuals. | ALL | ALL |
| 1. Ideally would want to provide consent to sharing personal health information before received the letter | CKD1, CKD3 | GP1, GP3 |
| 1. At least notification that you may receive a letter from this organization so this doesn’t come as a shock and you don’t question legitimacy of it; suggestions that this come from:  - The person who ordered the test (notify at the time of test ordering that they may get a letter) - Notice from the lab - At the time of renewing driver’s license / health card - A flyer about this that comes in the mail with CCO screening reminder letters - Ad campaign to launch this initiative | ALL | ALL |
| 1. Opt-in vs opt-out system  - Ideal if there was some type of advance notification about potentially receiving these letters and to allow you to opt-in to receive them rather than opt-out | - | GP2, GP3 |
| Involve the family MD/person who ordered the test from the start! | ALL | ALL |
| 1. Why is this letter not coming from whoever ordered the lab test? | CKD2, CKD3 | GP3 |
| 1. Why not target the doctors themselves?:  - “Why not go direct to the GP who ordered the lab result rather than direct to an individual, which suggests that you're bypassing the GP and potentially questioning the competency of the GP.” [CKD3 (R1 at 37:52)] - “This isn't somebody who's walking around with a brain aneurysm and doesn't know because nobody looked. If this is all based on test results that were ordered by a doctor then we do know, because somebody did look, so you should be going after those doctors that order those tests to follow up.” [GP2 (at 1:49:07)] | CKD3 | GP2 |
| 1. Copy the healthcare professional who ordered the test on all communications with the patient (a ‘cc’): “You've got to link the GP and the patient so that at least the GP knows that this may happen. The patient may get a letter, the patient may call the GP. There's got to be a whole communication mechanism around this initiative.” – [CKD3 (R1 at 1:43:28)] | CKD3 | GP2, GP3 |
| Concerns about getting bad news through a letter | CKD2, CKD3 | GP2, GP3 |
| 1. Bad news like this should be given in person or at least over the phone by a trusted healthcare professional (preferably family doctor)  - “Doesn’t make a lot of sense that this is the way you would be told.” [CKD2 (at 36:53)] | CKD2, CKD3 | GP2, GP3 |
| 1. Getting this kind of news in the mail can come at bad times in peoples’ days / lives – would prefer to login to a website that has this information instead when feeling ready to face bad news | - | GP2 |
| 1. In a letter, there would be nobody to explain what this means and help ease anxiety:  - “It's a really big deal to be told that you have kidney disease or potentially or in some stage of kidney failure. It's not an easy thing. I think a lot of people see it as a sentence on your life.” [CKD2 (R3 at 38:02)] - This comment also prompted us to tone down the message | CKD2 | - |
| Equity issue – across health conditions (one individual commented about this) | - | GP2 |
|  |  |  |
| **1.5 Response to the message** |  |  |
| Mixed emotions about the organization sending letters about this and how they got this information | ALL | ALL |
| 1. Anxiety (letter is alarming) | CKD2 | GP2 |
| - “As soon as I saw that box and I know this is only a focus group, but I didn't read the rest of the letter. I couldn't focus. I was just focused on that box. Oh no, my kidney. They found something with my kidney. I need more research. Oh, no. What's going to happen? What's the problem? And then I had a hard time reading the rest of the letter saying that they want to connect me with other services for further testing” – [GP2 (R2 at 36:36)] | - | GP2 |
| - “It would cause me great concern if I knew nothing about kidney disease and all of a sudden, I see physician name signed off. I don't know who these doctors are and they're telling me that I might be at risk. So it just feels isolated, out of context from my primary care.” [CKD3 (R4 at 38:58)] | CKD3 | - |
| 1. Grateful/ Hopeful |  |  |
| - May get care that otherwise would not have been received:   - “I would be thrilled that, oh, my gosh, somebody else is reaching out to me, especially up here in the Northwest, where medicine is much more difficult to get a hold of. I'd be thrilled to get an extra level of attention. And it would give me some hope and also the proactive feeling that was being projected into it.” [GP3 (R3 at 17:46)]   - If I was in, you know, emergency or somewhere like a walk-in clinic where it wasn't my own doctor and they sent me for tests and something came up. I'd really appreciate having these kind of things because not everybody has the family doctor.” – [GP1 (at 30:09)] | - | GP1, GP3 |
| - Someone is looking out for them:   - “I like the idea that someone out there is paying attention to my health. They're looking out for me and they're getting in touch with me. I might be too busy to do so, or I might not want to know. But because I'm receiving information now, it's all up to me to take the next step. So I think that's important.” - [CKD3 (at 2:09:38)]   [This was generally voiced by people who are not receiving high quality primary care (in many cases they do not have a family doctor)] | CKD3 | GP2, GP3 |
| 1. Anger (information shared without their consent) | CKD3 | GP2 |
| - “I would be livid to be approached like this. Who are these people? How did they get to see my medical records or test results without my permission, without my knowledge, without my consent? And I would think they were a bunch of creepy snoops, and I wouldn't want anything to do with them.” [GP2 (R6 at 38:17)] | - | GP2 |
| Interest in participating and would respond to letter | ALL | ALL |
| 1. Particularly for people from Northern Ontario / people without reliable primary care | - | ALL |
| 1. Health is very important to them: “I'm hooked because I'm concerned about my health” – [GP1 (at 45:24)] | CKD3 | ALL |
| 1. Recognition that some individuals may not be as proactive about health or have different life priorities (for e.g., low income) and may have different levels of responding | CKD2, CKD3 | - |
| 1. Prefer that this information comes from their doctor, but even if this is not their HCP that contacts them directly, would prefer to have this information rather than not | CKD2 | - |
| Almost everyone would check with their HCP before responding | ALL | ALL |
| 1. “I might have some alarm to say I did not know I had that condition. That might be a reaction to it. And a second thought is I might want to consult my family doctor before taking action.” – [GP1 (R4 at 44:16)] | - | GP1 |
| 1. Would take letter to family MD and ask them why they received this letter / follow what they recommend | ALL | ALL |
| Most would look more into the organizations (KCO and ICES) before responding | CKD1, CKD3 | GP2, GP3 |
| 1. Make sure key people at these organizations are aware of the study, in the event they are contacted. [Consider creating some notice on their websites advising of the study – even on the home page.] | CKD3 | - |
|  |  |  |
| **2 Acceptability of the outreach program overall** |  |  |
| Majority saw this as a positive program: | ALL | ALL |
| 1. Like the idea of catching people who have fallen through the cracks; prevention and earlier detection  - “And I'm sure most folks are familiar with people that they may know or may have heard of who were detected of a cancer situation which was Stage 4, right. So early detection saves lives or saves difficulty in dealing with whatever it is I'm hearing. A parallel here by being proactive to say that these lab tests say you're high at high risk, you have the opportunity. We've we detected it.” – [GP1 (at 1:13:38)] - “So I see this as the kidney care outreach as a good Plan B in case things fall through the cracks.” [GP2 (at 1:58:01)] | CKD2, CKD3 | GP1, GP2 |
| 1. Good for getting connected if have no family doctor or have uncoordinated care: “In the city where I live… we don't have a lot of doctors, so we rely on walk-in clinics. With the kidney care outreach, I would feel supported, I would be connected with counselors. I would be connected with support groups, everything I want to have with my family doctor and I don't. They would be offering it here in this specialized group in this specialized care directly for kidneys, and that would have me feeling supported, safe, heard, validated, that would give me what I need for that instance for what I'm going through.” – [GP2 (R2 at 2:10:20)] | CKD3 | GP2 |
| 1. An advantage would be connecting with virtual doctor – benefit those who live in more rural and remote areas: “Sometimes waiting for a specialist can take an incredibly long time. And for us that live in smaller areas that may not have as many specialists residing in your area, this is a good way to start the ball rolling.” - [GP2 (R5 at 1:54:28)] | - | GP2 |
| 1. Advantage of knowledge beforehand, can take steps needed: “Knowledge is power… knowing I can arrange my life so that I can make it as enjoyable as possible for whatever limited amount of time there might be” – [GP1 (at 2:25:03)] | - | ALL |
| Some changes needed for this to become an acceptable program and move beyond a pilot project | CKD2, CKD3 | ALL |
| 1. If this becomes the norm, we would be modifying healthcare in Ontario by taking responsibilities away from family providers, so society / family providers will need to be accepting of this:  - “You can probably get [family doctors] to buy into it much easier if you say this is intended to be potentially a province wide program which is intended to be supportive of you and the work that you do and not to take away from the MDs or the nurse practitioners, but a supporting mechanism for them. Because let's face it, the healthcare system today in Ontario is not in good shape. And we don't have enough doctors and there are an awful lot of people that don't have doctors. So this may be just one way of trying to provide that level of care that is currently not being provided at all.” – [CKD3 (R1 at 2:27:55)] | CKD3 | - |
| 1. Letters should come from an umbrella organization (i.e., Ontario government) that does this more broadly for a variety of conditions | CKD3 | GP1, GP2 |
| 1. More health information and awareness about seriousness of CKD is needed (unlike diabetes and heart disease which are more well known) | CKD2 | GP3 |
|  |  |  |
| **3 Expanding the model beyond kidney care** |  |  |
| **3.1 Interest regardless of severity of illness** |  |  |
| Moving beyond CKD, even if the condition was less severe or there was no treatment, people would still want to be notified about their risk for the following reasons: | ALL | ALL |
| 1. Knowledge of having this condition may still help them in some way (quality of life, can take steps needed to arrange life) | - | ALL |
| 1. Treatment may be available in the future / may benefit someone else (in regards to research) | - | GP2, GP3 |
| 1. Minority of people would not want to know (e.g., if older or no treatment) | CKD2 | GP2 |
|  |  |  |
| **3.2 Around using healthcare data to benefit Ontario citizens** |  |  |
| Theoretically should improve outcomes and reduce costs | ALL | ALL |
| 1. Good use of taxpayers’ money; collecting data is important | ALL | ALL |
| 1. Improves outcomes | CKD2 |  |
| Lack of improvement in healthcare system despite having this data | CKD1, CKD2 | GP1, GP3 |
| 1. “Keeping track of [healthcare data] is very useful as long as it's being used properly and not politically.” – [GP1 (at 1:03:37)] | - | GP1 |
| 1. Who makes the decisions on what data is extrapolated?  Concerned about how health decisions are being made in Canada. Is gathering this data going to make a difference? | CKD2 | - |
| Wish there was more information around use of healthcare data to monitor the healthcare system that is communicated to the public | GP2, GP3 | - |
| There is an ethical imperative to do outreach using this data: | ALL | ALL |
| 1. Would be unethical not to: “I think that there is an ethical imperative for sure and an obligation to let people know; if you don't know, you can't do anything if you know and you don't do anything. Well, OK, that's your choice. But at least you're given a choice.” – [CKD2 (R6 at 2:16:24)] | ALL | ALL |
| 1. Conflicted between privacy considerations and wanting to know this information/ doing the right thing | CKD2 | GP3 |
|  |  |  |
| **Other (does not fit into a theme but feels important)** |  |  |
| Some people check their lab test results online (so they may already be aware of these lab results) |  | GP2, GP3 |
